# Subchronic Intranasal Administration of NeuroEPO Reduces Long-Term Consequences of Severe Traumatic Brain Injury in Male Rats

**DOI:** 10.3390/antiox14060710

**Published:** 2025-06-11

**Authors:** Félix Iván López-Preza, Maria de los Angeles Nuñez-Lumbreras, Iliana Sosa-Testé, Alonso Fernández-Guasti, Luis Concha, Teresita Rodríguez-Obaya, Luisa Rocha

**Affiliations:** 1 Pharmacobiology Department, Center for Research and Advanced Studies of the National Polytechnic Institute (CINVESTAV), Mexico City C.P. 14330, Mexico; felix.lopez@cinvestav.mx (F.I.L.-P.); angeles_lumbreras@hotmail.com (M.d.l.A.N.-L.); jfernand@cinvestav.mx (A.F.-G.); 2 Center for the Production of Laboratory Animals (CENPALAB), Havana C.P. 10300, Cuba; iliana.sosa@cenpalab.cu; 3 Institute of Neurobiology, National Autonomous University of Mexico, Campus Juriquilla, Juriquilla C.P. 76230, Queretaro, Mexico; lconcha@unam.mx; 4 Center for Molecular Immunology (CIM), Havana C.P. 11600, Cuba; teresita@cim.sld.cu

**Keywords:** traumatic brain injury, NeuroEPO, sensorimotor function, oxidative stress, depression-like behavior, magnetic resonance imaging

## Abstract

Current treatments fail to prevent long-term consequences induced by a severe traumatic brain injury (TBI). This study aimed to evaluate the efficacy of repetitive intranasal administration of NeuroEPO (a derivative of erythropoietin) on long-term alterations after a severe TBI induced by the application of a lateral fluid percussion in male rats. A otal of 30–31 days after the trauma, TBI+vehicle group showed sensorimotor dysfunction (Neuroscore, *p* < 0.0009; beam walking test, *p* < 0.0001 vs. Sham+vehicle group) and depressive-like behavior suggested by increased immobility (*p* = 0.0009 vs. baseline) during the forced swim test. Rats also showed increased production of malondialdehyde (a marker of oxidative damage), increased catalase activity (an antioxidant enzyme), and atrophy of brain areas evaluated with Magnetic Resonance Imaging 31 days after the trauma. TBI+NeuroEPO group received intranasal administration of NeuroEPO (0.136 mg/kg) starting 3 h post-TBI and continued every 8 h for four days. This group showed less sensorimotor dysfunction (Neuroscore, *p* = 0.020; beam walking test, *p* = 0.001, vs. TBI+vehicle group) and normal immobility behavior (*p* = 0.998 vs. Sham+vehicle group). Levels of malondialdehyde and catalase as well as the volume of brain structures of this group were like the Sham+vehicle group. These findings support the potential of NeuroEPO as a therapeutic agent to reduce long-term consequences of TBI.

## 1. Introduction

Traumatic brain injury (TBI) is an alteration of the brain resulting from an external physical force such as a direct blow, penetrating wound, or explosive waves [1]. TBI is considered a silent epidemy in public health due to the high mortality and long-term disabilities experienced by survivors, which affect their daily lives. The complex pathophysiology of TBI is divided into two types: primary (occurring within seconds to hours after trauma) and secondary injuries (developing over days to weeks after trauma) [2,3]. Primary injuries include excessive release of glutamate [4], disruption of the blood–brain barrier [5], parenchymal hemorrhages [6] and axonal injuries [7]. The secondary injuries include neuroinflammation, neuronal death, hyperexcitability, and oxidative stress [8]. In this regard, the increase in the production of reactive oxygen species (ROS) is the main factor driving oxidative stress in the brain. Oxidative stress induces lipid peroxidation as consequence of ROS reaction with neuronal cell membranes. This process results in malondialdehyde (MDA) production, which is highly reactive, causing DNA damage and neuronal death [9]. Also, oxidative stress results in an imbalance in antioxidant enzymes, such as catalase (CAT) [10]. All these events may result in progressive brain atrophy, sensorimotor dysfunction, body weight changes and mood disorders [11,12,13,14].

In clinical practice, different pharmacological strategies have been tested to prevent long-term TBI complications [15]. One molecule studied for this purpose is erythropoietin (EPO) [15,16,17]. In animal models of TBI, EPO improves energy metabolism, reduces histopathological changes, and preserves the integrity of the blood–brain barrier [18,19]. However, it is currently not considered as a therapeutic agent for TBI because its chronic administration augments erythropoiesis and hemorrhages [20].

NeuroEPO is a derivative of EPO that differs from this molecule because of its low content of sialic acid. Due to this characteristic, NeuroEPO has low affinity for the EPO receptor and lacks erythropoietic effects [21]. Studies suggest that NeuroEPO is a promising therapeutic alternative for neurodegenerative disorders because it prevents excitotoxicity-induced cell death in vitro [22]. One limitation of NeuroEPO is its short serum half-life when administered systemically [23]. However, NeuroEPO achieves effective brain concentrations when applied intranasally [24], a route of administration that allows it to quickly penetrate the molecule into the brain and bypass the blood–brain barrier [25] without causing side effects in healthy volunteers [26].

Repeated intranasal administration of NeuroEPO to gerbils immediately after the induction of focal ischemia protects the brain from damage and reduces the neurological and cognitive dysfunctions [27]. In preclinical models of Alzheimer’s disease, intranasal administration of NeuroEPO has shown neuroprotective effects by maintaining mitochondrial integrity through the Bcl-2 factor and preventing the increase in the production of ROS [28]. Clinical studies revealed that the intranasal administration of NeuroEPO to patients with Alzheimer’s and Parkinson’s diseases slows cognitive declines and maintains stable hippocampal volume [29,30,31].

According to the previous group of evidence, we suggest that subchronic intranasal administration of NeuroEPO short-term after a severe TBI prevents long-term complications. For this purpose, we designed experiments to evaluate enduring changes induced by a severe TBI in animals receiving a subchronic intranasal administration of NeuroEPO starting 3 h after the trauma.

## 2. Materials and Methods

### 2.1. Animals

Male adult Wistar rats (250–300 g) were maintained individually in acrylic cages under controlled environmental conditions (12 h light/darkness cycles at 24 ± 1 °C and 50% humidity) with free access to food (LabDiet^®^ 5008) and tap water. All animal procedures and protocols were performed following the regulations established by the Mexican Official Norm (NOM-062-ZOO-1999) and with the approval of the Institutional Ethics Committee of the Center for Research and Advanced Studies of the National Polytechnic Institute (CICUAL project no. 0326-22).

### 2.2. Induction of Severe TBI

The lateral fluid percussion injury (LFPI) model was used to induce severe TBI [32]. Animals were anesthetized with ketamine (80 mg/kg, i.p.) and xylazine (17 mg/kg, i.m.) and placed in a stereotaxic frame. An incision was made following the sagittal suture, the periosteum was removed, and a 5 mm diameter craniotomy was performed on the left side (−5.0 mm to bregma and −4.0 mm to sagittal suture). A female Luer lock was fixed to the craniotomy using tissue glue, and a stainless-steel screw was implanted in the right frontal bone. Dental acrylic was used to fix the preparation to the skull.

TBI was induced after 90 min of deep anesthesia. Animals were connected to an LFPI device with a straight tip (AmScien Instruments, Model FP 302, Richmond, VA, USA) via the Luer lock, as described previously [32]. Then, a pulse pressure ranging between 2.8 and 3.6 atm was applied to induce severe TBI [33]. Immediately after TBI induction, animals were removed from the LFPI device, the acrylic helmet was removed, and the skin was sutured. Animals were then housed individually in acrylic cages on heating pads to maintain body temperature. Tramadol (20 mg/kg, s.c.) was administered 15 min after trauma to alleviate the pain induced by the experimental procedure.

### 2.3. Experimental Groups

Initially, the animals were habituated to intranasal administration as previously described [34]. Briefly, rats held in one hand received the intranasal administration of saline solution (5 µL/drops) during a period of 2 min, alternating drops between the left and right nostril (2 drops each nostril). The opposite nostril and mouth were maintained closed during this manipulation to facilitate the exposure of the nasal cavity to the vehicle. This procedure was repeated daily for 13 days. A total of 24 h after the last manipulation, rats were divided into 4 different groups.

**Figure 1:**
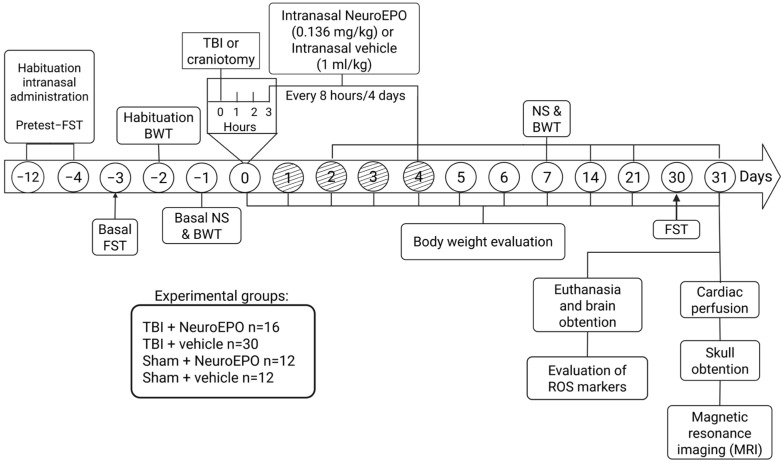
Timeline of the experimental design used to determine the effects of subchronic intranasal administration of NeuroEPO on body weight, sensorimotor function (NS and BWT), depression-like behavior (FST), ROS markers and brain volume (MRI) after a severe TBI. BWT: beam-walking test; CAT: catalase; DCFH-DA: dichlorofluorescein acetate; FST: forced swim test; MDA: malondialdehyde; NS: Neuroscore; ROS, reactive oxygen species; TBI, traumatic brain injury.

(a) TBI+NeuroEPO group (n = 16): Rats were submitted to severe TBI and received intranasal administration of NeuroEPO (0.136 mg/kg dissolved in PBS, at concentration of 1 mg/mL) 3 h after the trauma. NeuroEPO was obtained from the Center for Molecular Immunology (Havana, Cuba). Then, NeuroEPO administration was repeated every 8 h for 4 days following TBI. This treatment scheme has been reported to induce neuroprotection in other animal models [35,36]. Rats that died before receiving the first administration of NeuroEPO were not considered for mortality rate. Body weight, sensorimotor function and depressive-like behaviors were evaluated before and during 31 days after the trauma. On day 31, the surviving animals were divided into 2 groups: One group of rats was anesthetized with sodium pentobarbital (70 mg/kg, i.p.) and euthanized by decapitation. The brain was removed from the skull, and specific brain areas (cortex, hippocampus, cerebral amygdala, thalamus and hypothalamus) were dissected, immediately frozen and kept at −70 °C for the subsequent determination of oxidative stress markers. Another group of rats was anesthetized with sodium pentobarbital (70 mg/kg, i.p.) and perfused for the ex vivo evaluation of the volume of brain structures using magnetic resonance imaging (MRI) (Figure 1).

(b) TBI+vehicle group (n = 30): Rats were submitted to the same experimental procedure as the TBI+NeuroEPO group, except that they received intranasal administration of PBS (1 mL/kg) instead of NeuroEPO.

(c) Sham+NeuroEPO group (n = 12): Animals were manipulated as described for the TBI+NeuroEPO group, except that trauma was not induced.

(d) Sham+vehicle group (n = 12): Rats were submitted to the same experimental procedure as the Sham+NeuroEPO group, except that they received intranasal administration of PBS (1 mL/kg) instead of NeuroEPO.

### 2.4. Evaluation of Sensoriomotor Function

Sensorimotor function was evaluated with 2 different procedures:

(a) The Neuroscore (NS) includes a battery of four functional tests: (1) grasping ability on an inclined plane at different angles (from 60° to 75°); (2) resistance to a lateral pulsion (left–right); (3) forelimb and (4) hindlimb extension and counter flexion. Each test score ranged from 0 points, when the function was completely lost, to 4 points for normal function. A final score of 26 to 28 points indicates normal sensorimotor function, 20 to 25 points suggest mild dysfunction, 16 to 19 points indicate moderate dysfunction, and a score equal to or less than 15 points specifies severe dysfunction [37].

(b) The beam-walking test (BWT) was used to assess the motor coordination and balance of rats. Animals were habituated to this test before the surgery. In this test, rats crossed a beam (140 cm long and 12 cm wide located 43 cm above the ground) and moved towards a safety box. Motor function is scored as 6 points when the animal is able to cross the beam without foot-slips (normal function); 5 points when the rat crosses the beam with at least 3 foot-slips; 4 points when the animal shows 6 or more foot-slips; 3 points when the rat presents an affected limb that avoids forwarding locomotion; 2 points if the animal falls down while walking on the beam; 1 point when the rat is unable to cross the beam but does not fall down; and 0 points when the animal falls down without walking across the beam. The BWT was applied over 3 consecutive trials per session [38]. A low score obtained from the average of the 3 trials indicated impairment of the motor function.

NS and BWT were assessed under basal conditions 24 h before the craniotomy and then on days 2, 7, 14, 21, and 31 after trauma or manipulation. Both tests were performed as a double-blind trial to reduce bias.

### 2.5. Evaluation of Depression-like Behaviors

The forced swim test (FST) is used to screen drugs with putative antidepressant-like effects. In this study, FST was used to identify behaviors that reflect depression-like behavior [39]. The test consists of introducing the rat into a transparent glass cylinder container (46 cm tall, 20 cm in diameter) filled with water to a height of 30 cm, and maintained at 24 ± 2 °C. A pre-test was carried out for 15 min for habituation between day 4 and day 12 before the TBI or craniotomy. Then, the animals were submitted to the test, conducted for 5 min, at 72 h before the craniotomy (basal FST) and on day 30 after trauma or manipulation (experimental FST). The behavior during basal and experimental FST was recorded using a video camera. The videos were divided into segments of 5 s duration (“counts”). The number of “counts” during which immobility, swimming or climbing behaviors were shown were quantified [40]. The counts obtained from basal and experimental FST were compared.

### 2.6. Evaluation of Oxidative Stress Markers in Brain Structures

Oxidative stress markers were evaluated in brain areas (cortex, hippocampus and cerebral amygdala (ipsi- and contralateral to the craniotomy), thalamus and hypothalamus) obtained from euthanized rats. The tissue was homogenized on dry ice using a solution containing protease inhibitors (cOmplete™ Mini EDTA-free Protease Inhibitor Cocktail; Cat. No. 4693159001; Roche, Mannheim, Germany) and supplemented with phosphatase inhibitors (PhosSTOP EASYpack, phosphatase inhibitor cocktail; No. 4906845001, Roche, Mannheim, Germany). Following homogenization, the samples underwent centrifugation at 14,000 rpm for 10 min at 4 °C. Total protein content was determined using the Bradford assay (Quick Start™ Bradford Protein Assay Kit 1; Cat. No 5000201; Bio-Rad; Hercules, California; USA) according to the manufacturer’s instructions. The homogenates were divided into 3 fractions for the subsequent evaluations.

(a) Estimation of ROS production

Dichlorofluorescein was used as a non-specific marker that allows the quantification of intracellular ROS [41]. A fraction of the homogenized tissue (1 μL) was added to 195 μL of TRIS:HEPES (18:1) and incubated in the presence of 25 μL of 2′,7′-dichlorofluorescein diacetate (5 μM) for 1 h at 37 °C. ROS in the sample interact with non-fluorescent 2′,7′-dichlorofluorescein diacetate and form fluorescent 2,7-dichlorofluorescein (DCF). The fluorescence was measured in a spectrophotometer (BioTek FLx800, Winoosky, Vermont, USA) at an excitation wavelength of 485 nm and an emission wavelength of 528 nm. DCF concentrations were determined using a linear regression analysis based on an external standard calibration curve (0, 1, 2, 4, 8, 16, 32, 40, 60, 80, 100, and 200 ng/μL) of DCF. The results were expressed as nanograms of DCF formed per milligram of protein per hour.

(b) Analysis of malondialdehyde production

MDA is a byproduct of lipid peroxidation and is used as a marker of oxidative stress [42]. A fraction of the homogenized tissue (20 μL) was added to 20 μL of 10% trichloroacetic acid solution and 300 μL of TBA solution. Thereafter, the reaction was incubated in a boiling water bath for 1 h and then placed on ice to stop it. Then, 100 μL of the reaction was added in duplicate to a 96-well plate for subsequent reading on the spectrophotometer at 549 nm (Multiskan FC; SN 357-714338, Thermo scientific; Woodlands, Singapore; Shanghai, China). MDA production was determined using a linear regression analysis based on an external standard calibration curve (0.625, 1.25, 2.5, 5, 10, 25, 50, and 100 μM) of MDA. The results were expressed as nanomoles of MDA formed per milligram of protein.

(c) Evaluation of catalase activity

CAT is an antioxidant enzyme responsible for breaking down hydrogen peroxide (H_2_O_2_) into water and oxygen, thereby mitigating oxidative stress [43]. The activity of CAT was evaluated using a method previously described with some modifications [44]. A fraction of the homogenized tissue (20 μL) was mixed with 1 mL of phosphate buffer (pH 7.4) and 2 mL of 30% hydrogen peroxide solution. CAT-mediated decomposition of H_2_O_2_ was measured spectrophotometrically at 240 nm for 2 min using a Jenway 6715 UV/Visible spectrophotometer (Serial No. 37174, Stone, UK). The results were expressed as units per milliliter per milligram of protein, calculated using a mathematical formula previously reported [45].

### 2.7. Analysis of Brain Volume by Ex Vivo MRI

Reduced volume of the brain suggests cerebral damage. The animals were perfused intracardially with 250 mL of saline solution (0.9%) containing heparin (1 mg/L, Sigma-Aldrich, Cat # H3393) and gadolinium-based contrast agent (2 mM, Prohance, Bracco Diagnostics Inc; Singen; Wurtemberm Germany), followed by 250 mL of PBS containing paraformaldehyde (4%, Sigma-Aldrich Cat # P6148), glutaraldehyde (0.2%, Electron Microscopy Sci. Cat # 16210; Hatfield, PA) and Prohance (2 mM). After perfusion, the head was removed from the body and submerged in a solution of 4% paraformaldehyde and 2 mM ProHance at 4 °C overnight. The next day, the solution was replaced with PBS containing 0.02% sodium azide (Sigma-Aldrich, Cat # S2002) and 2 mM ProHance. The samples were kept at 4 °C until imaging. MRI was performed using a 7.0 T magnet, with an acquisition time of 1 h per sample, a resolution of 85 µm, a repetition time of 30 ms, an echo time of 8.6 ms at 20 °C. The volumetric analysis of individual brain structures was manually performed using a rat brain atlas and the ITK-SNAP software version 4.2.0. Individual masks were delineated to include the voxels corresponding to the brain areas ipsilateral and contralateral to the TBI or craniotomy. The selected voxels were summated to obtain the total volumes.

### 2.8. Statistical Analysis

The results were expressed as the means ± standard error. A two-way repeated-measures analysis of variance followed by Tukey’s post hoc test was used to analyze the values obtained from body weight and sensorimotor function evaluation. Sidak’s post-hoc test was used for the results obtained from the FST. Two-way analysis of variance and Tukey’s post hoc test were used to analyze the REDOX environment. One-way analysis of variance and Tukey’s post hoc test were applied to analyze the structures’ brain volume results. The statistical analysis and figure design were performed with GraphPad Prism 10.0.1 (GraphPad Software, San Diego, CA, USA). Statistical significance was accepted at *p* < 0.05.

## 3. Results

### 3.1. Intranasal Administration of NeuroEPO Lessens Mortality Rate and Improves Body Weight Gain and Sensorimotor Recovery After a Severe TBI

No mortality was found in the Sham+vehicle and Sham+NeuroEPO groups. In contrast, the TBI+vehicle group had a mortality rate of 60% (18 died and 12 survived, *p* < 0.0001 vs. Sham+vehicle group). The TBI+NeuroEPO group had a lower mortality rate (25%, 4 died and 12 survived, *p* = 0.010 vs. TBI+vehicle group). According to these results, all experimental groups had a total of 12 animals each 30 days after TBI or manipulation.

A two-way RM ANOVA of body weight changes revealed significant effects of Time × Group interaction (F_(30, 440)_ = 1.849, *p* = 0.004), Time (F_(1.687, 74.24)_ = 349.3, *p* < 0.0001), Group (F_(3, 44)_ = 9.336, *p* < 0.0001), and Subject (F_(44, 440)_ = 5.700, *p* < 0.0001).

The Sham+vehicle group showed a decrease in body weight up to 4.95 ± 1.36% on day 2 post-craniotomy, followed by a gradual recovery achieving a gain of 44.59 ± 2.75% on day 31 after surgery. The Sham+NeuroEPO group showed a significant decrease in body weight on day 1 post craniotomy (7.99 ± 0.65%, *p* = 0.003 vs. Sham+vehicle group). Thereafter, animals gradually regained weight, achieving values similar to the Sham+vehicle group on day 31 post-craniotomy (52.48 ± 6.33%, *p* = 0.6701).

**Figure 2:**
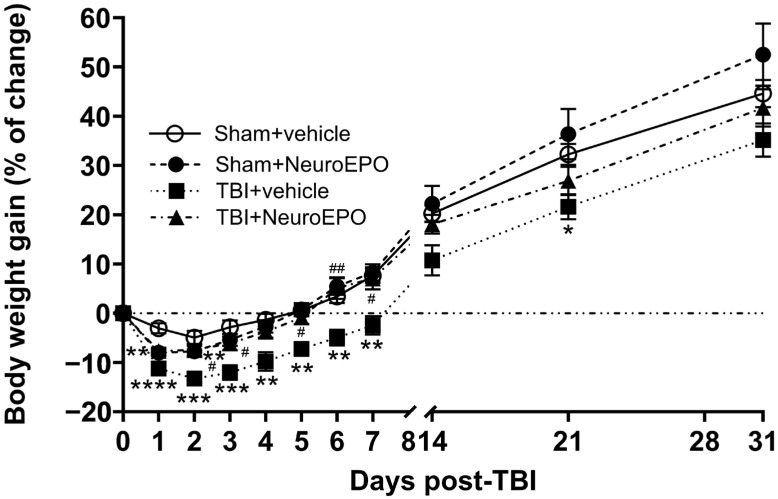
Effect of subchronic intranasal administration of NeuroEPO or vehicle on the gain in body weight through 31 days after severe TBI or manipulation. Values represent the body weight gain expressed in percentage of change in relation to the weight recorded at day 0 before the craniotomy. Each bar represents the mean ± standard error of 12 animals. Asterisks (*) denote significant differences vs. Sham+vehicle group. Hash symbols (#) denote significant differences vs. TBI+vehicle group. Statistical analysis was performed using Two-Way RM ANOVA, followed by Tukey’s post hoc test. * *p* < 0.05; ** *p* < 0.01; *** *p* < 0.001; **** *p* < 0.0001; # *p* < 0.05; ## *p* < 0.01. RM: repeated-measures.

The TBI+vehicle group showed a significant decrease in body weight 2 days after trauma (13.24 ± 1.13%, *p* = 0.0007 vs. Sham+vehicle group), followed by a gradual recovery. However, the body weight of this group was lower throughout the experimental procedure (Figure 2). The body weight of TBI+NeuroEPO group was decreased on day 1 after TBI (7.67 ± 0.68%, *p* = 0.006 vs. Sham+vehicle group). Thereafter, rats from this group gradually regained and achieved values similar to the Sham+vehicle group at day 31 post-TBI (41.69 ± 3.78%, *p* = 0.924) (Figure 2).

Concerning sensorimotor function, two-way RM ANOVA for NS and BWT revealed significant effects of Time × Group interaction (NS: F_(15, 220)_ = 20.01, *p* < 0.0001; BWT: F_(15, 220)_ = 21.90, *p* < 0.0001), Time (NS: F_(3.214, 141.1)_ = 67.75, *p* < 0.0001; BWT: F_(3.761, 165.5)_ = 73.15, *p* < 0.0001), Group (NS: F_(3, 44)_ = 40.09, *p* < 0.0001; BWT: F_(3, 44)_ = 79.69, *p* < 0.0001), and Subject (NS: F_(44, 220)_ = 7.648, *p* < 0.0001; BWT: F_(44, 220)_ = 3.639, *p* < 0.0001).

**Figure 3:**
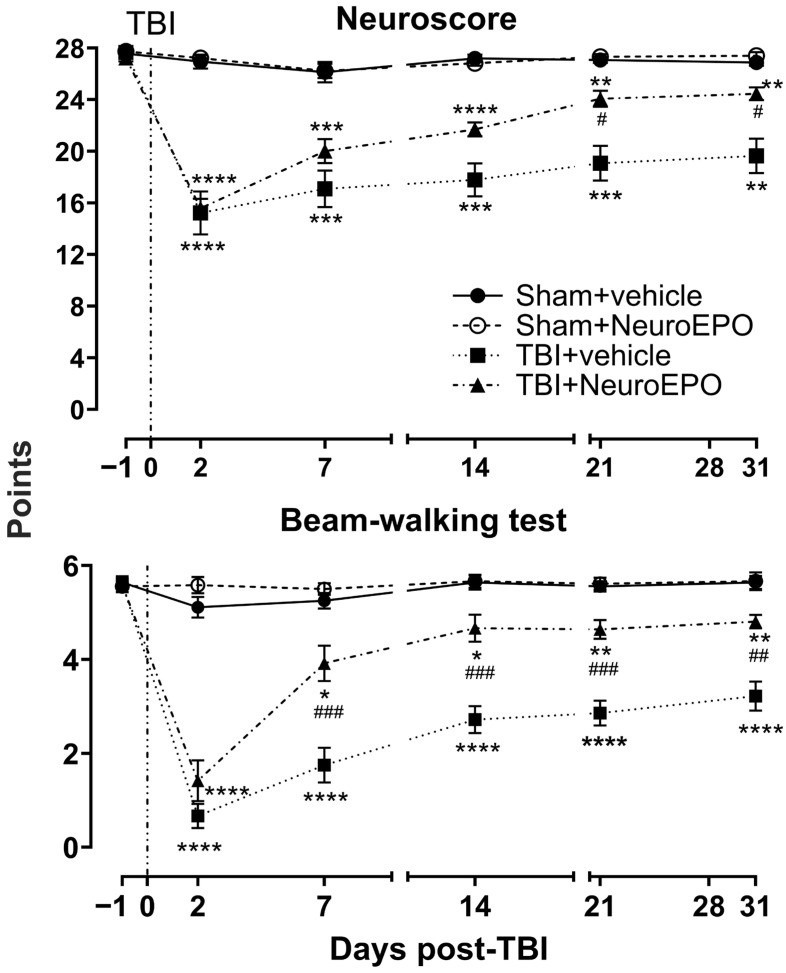
Effect of subchronic intranasal administration of NeuroEPO or vehicle on sensorimotor function through 31 days after severe TBI or manipulation. Sensorimotor function was evaluated using Neuroscore battery (upper graph) and beam-walking test (lower graph). Each value represents the mean ± standard error of 12 animals. Asterisks (*) indicate significant differences vs. Sham+vehicle group. Hash symbols (#) indicate significant differences vs. TBI+vehicle group. Statistical analysis was performed using Two-Way RM ANOVA followed by Tukey’s post hoc test. * *p* < 0.05; ** *p* < 0.01; *** *p* < 0.001; **** *p* < 0.0001; # *p* < 0.05; ## *p* < 0.01; ### *p* < 0.001. RM: repeated-measures.

The Sham+vehicle and Sham+NeuroEPO groups obtained values in the normal range throughout the experimental procedure (Sham+vehicle group: 27.58 ± 0.22 for NS and 5.63 ± 0.13 for BWT; Sham+NeuroEPO group: 27.75 ± 0.22 for NS and 5.55 ± 0.12 for BWT). The TBI+vehicle group’s scores decreased on day 2 post-TBI (NS, 44.85% lower, *p* < 0.0001; BWT, 88.23% lower, *p* < 0.0001 vs. Sham+vehicle group). Although the sensorimotor function progressively improved, rats from the TBI+vehicle group presented a moderate dysfunction 31 days after the trauma (NS, 28.87% lower, *p* = 0.0009; BWT, 42.60% lower, *p* < 0.0001 vs. Sham+vehicle group). TBI+NeuroEPO group showed decreased sensorimotor function on day 2 post-TBI (NS, 42.82% lower, *p* < 0.0001; BWT, 74.73% lower, *p* < 0.0001 vs. Sham+vehicle group). However, rats from this group showed improved sensorimotor function classified as mild, 31 days after the trauma (*p* = 0.020 and *p* = 0.001 for NS and BWT, respectively, vs. TBI+vehicle group) (Figure 3).

### 3.2. Intranasal Administration of NeuroEPO Reduces Depression-like Behavior Induced by Severe TBI

The basal immobility times recorded in the FST did not show significant differences among the experimental groups (F_(3,44)_ = 0.250, *p* = 0.086). The two-way RM ANOVA revealed a statistically significant effect of time (F_(1,44)_ = 9.114, *p* = 0.004), treatment (F_(3,44)_ = 2.909, *p* = 0.045), and their interaction (F_(3,44)_ = 17.22, *p* < 0.0001).

**Figure 4:**
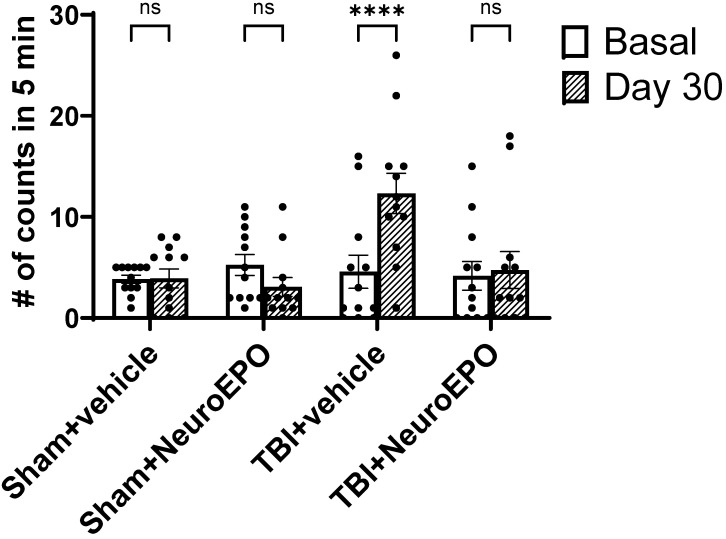
Effect of subchronic intranasal administration of NeuroEPO or vehicle on immobility (a depression-like behavior) during the FST under basal conditions and 30 days after a severe TBI or manipulation. Each dot represents the value obtained from an individual animal. Each bar represents the mean ± standard error of 12 animals. Asterisks (*) indicate significant differences compared to baseline values of the same group. Statistical analysis was performed using Two-Way RM ANOVA, followed by Sidak’s post hoc test. **** *p* < 0.0001. RM: repeated-measures. ns = not significant.

Rats from the TBI+vehicle group presented an increase in immobility 31 days after trauma (*p* < 0.0001 vs. basal values) whereas TBI+NeuroEPO group showed levels of immobility like the Sham+vehicle group (Figure 4).

The increase in immobility observed in TBI+vehicle group occurred at the expense of a decreased swimming behavior.

### 3.3. Intranasal Administration of NeuroEPO Avoids TBI-Induced REDOX Imbalance in Different Brain Areas

Concerning the evaluation of ROS production with dichlorofluorescein, a two-way ANOVA of ROS production across brain structures revealed the following results: group × hemisphere interaction (Cortex: F_(3,40)_ = 0.250, *p* = 0.860; hippocampus: F_(3,40)_ = 0.252, *p* = 0.859; amygdala: F_(3,40)_ = 0.201, *p* = 0.894), group effect (Cortex: F_(1,40)_ = 1.910, *p* = 0.174; hippocampus: F_(1,40)_ = 0.07, *p* = 0.792; amygdala: F_(1,40)_ = 0.006, *p* = 0.934) and main effect of side (Cortex: F_(3,40)_ = 14.87, *p* < 0.0001; hippocampus: F_(3,40)_ = 26.08, *p* < 0.0001; amygdala: F_(3,40)_ = 15.09, *p* < 0.0001). In case of thalamus and hypothamus, a one-way ANOVA of ROS production revealed significant differences among experimental groups (Thalamus: F_(3,4)_ = 31.47, *p* = 0.003; hypothalamus F_(3,20)_ = 15.47, *p* < 0.0001).

The Sham+vehicle and Sham+NeuroEPO groups showed similar values (cortex: ipsi-, *p* = 0.856, contra-, *p* = 0.984; hippocampus: ipsi-, *p* = 0.918, contra-, *p* = 0.998; amygdala: ipsi-, *p* > 0.999, contra, *p* = 0.902; thalamus: *p* = 0.648; hypothalamus: *p* = 0.867). The TBI+vehicle group presented a significant increase in ROS production compared to Sham+vehicle group: cortex (ipsi-, 295.17%, *p* = 0.002; contra-, 247.43%, *p* = 0.0003); hippocampus (ipsi-, 266.4%, *p* < 0.0001; contra-, 214.20%, *p* < 0.0001); amygdala (ipsi-, 217.25%, *p* = 0.002; contra-, 254.73%, *p* = 0.0009; thalamus (582.80%, *p* < 0.0001); and hypothalamus (564.23%, *p* = 0.001). The TBI+NeuroEPO group showed values similar to the Sham+vehicle group in cortex (ipsi-, 32.76%, *p* = 0.974; contra-, 33.80%, *p* = 0.923), hippocampus (ipsi-, 53.18%, *p* = 0.713; contra-, 8.91%, *p* = 0.995), contralateral amygdala (163.56%, *p* = 0.052), thalamus (146.96%, *p* = 0.422) and hypothalamus (184.02%, *p* = 0.516). However, rats from the TBI+NeuroEPO group exhibited increased ROS expression in the amygdala ipsilateral to the trauma (169.96%, *p* = 0.023 vs. Sham+vehicle) (Table 1).

Concerning MDA production, a two-way ANOVA of MDA production across brain structures revealed the following results: group × hemisphere interaction (cortex: F_(3,40)_ = 3.164, *p* = 0.034; hippocampus: F_(3,40)_ = 10.67, *p* < 0.0001; amygdala: F_(3,40)_ = 1.064, *p* = 0.375), group effect (cortex: F_(1,40)_ = 13.22, *p* = 0.0008; hippocampus: F_(1,40)_ = 9.794, *p* = 0.003; amygdala: F_(1,40)_ = 3.661, *p* = 0.062) and main effect of side (cortex: F_(3,40)_ = 85.52, *p* < 0.0001; hippocampus: F_(3,40)_ = 108.2, *p* < 0.0001; amygdala: F_(3,40)_ = 20.84, *p* < 0.0001). In the case of thalamus and hypothamus, a one-way ANOVA of MDA production revealed significant differences among experimental groups (Thalamus: F_(3,20)_ = 14.89, *p* < 0.0001; hypothalamus F_(3,20)_ = 11.28, *p* = 0.0002).

The Sham+vehicle and Sham+NeuroEPO groups showed similar MDA production (cortex: ipsi-, *p* = 0.999, contra-, *p* = 0.990; hippocampus: ipsi-, *p* = 0.979, contra-, *p* = 0.725; amygdala: ipsi-, *p* = 0.773, contra, *p* = 0.999; thalamus: *p* = 0.570 and hypothalamus: *p* = 0.475). In contrast to the Sham+vehicle group, the TBI+vehicle group showed a significant increase in MDA production in all the brain areas evaluated: cortex (ipsi-, 230.73%, *p* < 0.0001; contra-, 182.76%, *p* < 0.0001), hippocampus (ipsi-, 462.36%, *p* < 0.0001; contra-, 203.57%, *p* < 0.0001), amygdala (ipsi-, 194.29%, *p* < 0.0001; contra-, 119.38%, *p* = 0.005), thalamus (193.14%, *p* < 0.0001) and hypothalamus (332.03%, *p* = 0.0001). The values obtained from the TBI+NeuroEPO group were similar to the Sham+vehicle group in the different brain structures estimated (cortex: ipsi-, 13.47%, *p* = 0.9126; contra-, −4.35%, *p* = 9979; hippocampus: ipsi-, −0.52%, *p* < 0.999; contra-, 5.29%, *p* = 0.9976; thalamus: 37.76%, *p* = 0.6304 and hypothalamus: 87.80%, *p* = 0.4910), except amygdala, which showed increased MDA production when compared with the Sham+vehicle group (ipsi-, 194.86%, *p* < 0.0001; contra-, 91.33%, *p* = 0.045) (Table 1).

Concerning CAT activity, a two-way ANOVA of CAT activity across brain structures revealed the following results: group × hemisphere interaction (cortex: F_(3,40)_ = 0.074, *p* = 0.973; hippocampus: F_(3,40)_ = 0.135, *p* = 0.938; amygdala: F_(3,40)_ = 1.206, *p* = 0.319), group effect (cortex: F_(1,40)_ = 0.045, *p* = 0.831; hippocampus: F_(1,40)_ = 0.414, *p* = 0.523; amygdala: F_(1,40)_ = 0.083, *p* = 0.774) and main effect of side (cortex: F_(3,40)_ = 14.85, *p* < 0.0001; hippocampus: F_(3,40)_ = 8.108, *p* = 0.0002; amygdala: F_(3,40)_ = 14.31, *p* < 0.0001). In the case of thalamus and hypothamus, a one-way ANOVA of CAT activity revealed significant differences among experimental groups (Thalamus: F_(3,20)_ = 10.57, *p* = 0.0002; hypothalamus F_(3,20)_ = 24.39, *p* < 0.0001).

The Sham+vehicle and Sham+NeuroEPO groups showed similar values (cortex: ipsi-, *p* = 0.999, contra-, *p* = 0.992; hippocampus: ipsi-, *p* = 0.980, contra-, *p* = 0.997; amygdala: ipsi-, *p* = 0.877, contra, *p* > 0.999; thalamus: *p* > 0.999 and hypothalamus: *p* = 0.999). When compared with Sham+vehicle group, the TBI+vehicle group showed a significant increase in CAT activity in cortex (ipsi-, 720.35%, *p* = 0.001; contra-, 459.3%, *p* = 0.007), hippocampus (ipsi-, −241.11%, *p* = 0.017; contra-, 228.08%, *p* = 0.083), amygdala (ipsi-, 204.61%, *p* = 0.0003; contra-, 121.62%, *p* = 0.040), thalamus (612.1%, *p* < 0.0001) and hypothalamus (699.6%, *p* < 0.0001). The different brain areas of the TBI+NeuroEPO group showed a CAT activity similar to the Sham+vehicle group (cortex: ipsi-, −3.76%, *p* > 0.999; contra-, −38.84%, *p* = 0.991; hippocampus: ipsi-, 11.08%, *p* > 0.999; contra-, 12.82%, *p* > 0.999; amygdala: ipsi-, 86.73%, *p* = 0.231; contra-, 97.23%, *p* = 0.1360; thalamus: 19.58%, *p* = 0.998 and hypothalamus: 95.6%, *p* = 0.7513) (Table 1).

### 3.4. Intranasal Administration of NeuroEPO Prevents TBI-Induced Brain Atrophy

The analysis of MRI images showed significant volumetric changes across brain regions, according to one-way ANOVA in cortex ipsi (F_(3,17)_ = 10.52, *p* = 0.0004), contra (F_(3,17)_ = 7.393, *p* = 0.0022), hippocampus ipsi (F_(3,4.88)_ = 0.425, *p* = 0.042), contra (F_(3,17)_ = 3.447, *p* = 0.040); amygdala ipsi (F_(3,17)_ = 5.624, *p* = 0.0073), contra (F_(3,17)_ = 5.859, *p* = 0.006); thalamus (F_(3,17)_ = 11.43, *p* = 0.0002) and hypothalamus (F_(3,17)_ = 7.696, *p* = 0.0018). The Sham+vehicle and Sham+NeuroEPO groups presented similar volume values for the different brain areas evaluated (cortex: ipsi-, *p* = 0.518, contra-, *p* = 0.212; hippocampus: ipsi-, *p* = 0.981, contra-, *p* = 0.877; amygdala: ipsi-, *p* = 0.998, contra, *p* = 0.903; thalamus: *p* = 0.650; hypothalamus: *p* > 0.999). In contrast to the Sham+vehicle group, the TBI+vehicle group showed a significant reduction in the volume of the cortex (ipsi-, −23.4%, *p* = 0.001; contra-, −32.2%, *p* = 0.001), ipsilateral hippocampus (−29.5%, *p* = 0.0009), amygdala (ipsi-, −18.4%, *p* = 0.041; contra-, −19.8%, *p* = 0.023), thalamus (−26.7%, *p* = 0.044) and hypothalamus (−17.1%, *p* = 0.007). The TBI+NeuroEPO group showed values similar to the Sham+vehicle group (cortex: ipsi-, −1.60%, *p* = 0.988; contra-, −13.7%, *p* = 0.245; hippocampus: ipsi-, 4.96%, *p* = 0.446; contra-, −21.8%, *p* = 0.290; amygdala: ipsi-, 0.83%, *p* = 0.997; contra-, −2.9%, *p* = 0.882; thalamus: 19.1%, *p* = 0.224; hypothalamus: −2.14%, *p* = 0.966) (Table 2).

## 4. Discussion

The present research supports previous studies indicating that severe TBI induces long-term sensorimotor dysfunction, low body weight gain and mood disorders associated with a high mortality rate, REDOX imbalance and brain damage [13,32,46,47,48,49]. Our experiments revealed that the subchronic intranasal administration of NeuroEPO after severe trauma prevents the long-term REDOX imbalance and brain atrophy. These results were associated with a better sensorimotor performance, improved body weight gain, and lower expression of depressive-like behaviors.

In normal (non-injured) rats, the subchronic intranasal administration of NeuroEPO did not induce changes in the different parameters evaluated. These results agree with previous reports indicating that the intranasal administration of EPO analogues with low sialic acid levels does not induce side effects in healthy animals [50] and humans [26].

The high mortality rate after severe TBI may be due to damage to the brainstem [51]. The brainstem is responsible for various functions that are disrupted after a TBI, such as cardiovascular and respiratory control, several autonomic functions, among others, ultimately leading to the patient’s death [51,52,53]. In addition, our experiments support that severe TBI results in a long-term REDOX imbalance leading to lipid peroxidation driven by excessive ROS expression, forming highly reactive pro-oxidants such as MDA and 4-hydroxynonenal [54]. On the other hand, increased activity of CAT, an important antioxidant enzyme, is detected in the short [55,56] and long term after severe TBI (present study). However, this brain’s natural antioxidant defense system is insufficient to prevent damage [57]. The long-term REDOX imbalance and brain atrophy found in the present study and previous reports [14] can lead to several dysfunctions detected in the long term after severe TBI. Concerning this issue, lower body weight gain after a severe TBI [4,58] can be associated with the damage of the hypothalamus [59], dysfunction of the enteric system [60] and hypermetabolism [61]. The sensorimotor dysfunction post-TBI may result from the injury of the cortex and thalamus, brain regions involved in sensorimotor integration [62]. Likewise, post-TBI depressive-like behavior found in preclinical models has been associated with the injury of the cerebral amygdala and hippocampus [63,64].

The results of the present study indicate that the subchronic intranasal administration of NeuroEPO starting 3 h after a severe TBI reduces the long-term consequences evaluated in our different experiments. The neuroprotective effects of this therapy have been replicated in preclinical and clinical studies of neurodegenerative diseases. For example, intranasal administration of NeuroEPO 3 times per week for 48 weeks or once per week for 5 weeks improved the cognitive functions of patients with mild to moderate Alzheimer’s disease and patients with Parkinson’s disease, respectively [29,31,65]. In preclinical studies, repeated intranasal administration of NeuroEPO 3 times a day for 4 days induced neuroprotection in a focal ischemia model [35].

Mood disorders are common consequences in patients with severe TBI [11]. Indeed, the high incidence of depression following a TBI (77%) is associated with disabling physical symptoms, lower quality of life, and decreased psychosocial functioning [66]. The results obtained from the present study revealed that the intranasal administration of NeuroEPO prevents depressive-like behaviors. This is the first report suggesting that intranasal NeuroEPO administration after severe trauma could be a good therapy to avoid mood disorders.

TBI causes brain atrophy [14] due to neuronal death provoked by increased oxidative stress and apoptosis, among others [67]. Our results indicate that intranasal administration of NeuroEPO prevents the reduction in brain structure volume. The neuroprotective effects of NeuroEPO are associated with its antioxidant and anti-apoptotic effects [22,27,68]. Concerning this issue, it is known that the activation by NeuroEPO of the heteroreceptor composed of EPOR and the receptor β-common subunit upregulates Bcl-2 and reduces the glutamate-induced activation of caspase-3 with a consequent reduction in the production of apoptotic cells [22,69]. NeuroEPO also decreases oxidative stress and lipid peroxidation by activating the PI3K/Akt pathway [36]. It is also indicated that NeuroEPO promotes the expression of neuroglobin in damaged cerebral regions, which would result in the restoration of brain homeostasis [70].

The intranasal route of administration of NeuroEPO may facilitate its penetration into the brain in a short time through the olfactory bulb and trigeminal nerve [71]. The absorption of NeuroEPO through the olfactory bulb can facilitate its axonal transport to brain areas such as the amygdala and its diffusion to other brain structures through the extracellular space [71]. Through the trigeminal nerve, NeuroEPO can reach the brainstem [72] and reduce cardiovascular and respiratory dysfunctions after severe TBI, thereby reducing mortality. However, future studies are essential to support these ideas.

We found that the repeated intranasal administration of NeuroEPO for 4 days did not prevent the TBI-induced changes in the expression of oxidative stress markers in the cerebral amygdala. It is known that the glucose metabolism of the cerebral amygdala is high under basal conditions [73]. If TBI augments the brain glucose metabolism [74], it is possible that higher doses and/or prolonged treatment with NeuroEPO are essential to prevent the high ROS expression in the amygdala.

A limitation of the present study is the lack of evaluation of NeuroEPO effects in females. Additionally, memory tests are needed to provide a more detailed assessment of hippocampal status and the effects of NeuroEPO administration after TBI. Future studies are essential to determine whether NeuroEPO is able to avoid post-TBI damage in brain areas such as the brainstem and reduce the mortality after trauma.

## 5. Conclusions

NeuroEPO has therapeutic potential for addressing the complex aftermath of severe TBI. These findings underscore the importance of exploring innovative treatments for severe TBI and highlight the neuroprotective properties of NeuroEPO when applied by intranasal route. These results might offer hope for recovery and improved quality of life for individuals affected by severe TBI using a therapy of easy and safe administration.

## Figures and Tables

**Table 1:** Effects of the intranasal administration of NeuroEPO on reactive oxygen species (ROS), catalase activity and malondialdehyde levels in brain homogenates of rats submitted to severe TBI.

Markers	Experimental Groups	Side	Cerebral Structures				
			Cortex	Hippocampus	Amygdala	Thalamus	Hypothalamus
DCF (ng DCF formed/mg of protein/h	Sham+vehicle n = 6	Ipsi-	2822 ± 578.8	3092 ± 549.9	4390 ± 2010	1963 ± 226.3	1565 ± 333.8
		Contra-	4044 ± 756.3	3903 ± 847.9	4055 ± 1641		
	Sham+NeuroEPO n = 6	Ipsi-	4561 ± 1562	4080 ± 972.3	4262 ± 1809	3393 ± 1204	3993 ± 1594
		Contra-	4824 ± 1218	3633 ± 725.8	5718 ± 2153		
	TBI+vehicle n = 6	Ipsi-	11,150 ± 1253 **	11,331 ± 1267 ****	13,928 ± 1899 **	3393 ± 1204 ***	10,392 ± 1248 ****
		Contra-	14,051 ± 3171 ***	12,272 ± 1754 ****	14,384 ± 1588 ***		
	TBI+NeuroEPO n = 6	Ipsi-	3746 ± 976.2 ^##^	4737 ± 1142 ^###^	11,851 ± 865.7 *	4863 ± 1873 ^#^	4444 ± 2076 ^###^
		Contra-	5411 ± 1331 ^##^	4251 ± 1040 ^####^	10,687 ± 1810		
Catalase (U/mL/mg of protein)	Sham+vehicle n = 6	Ipsi-	3.72 ± 1.20	7.36 ± 1.91	13.42 ± 2.10	5.26 ± 0.80	5.48 ± 1.94
		Contra-	4.90 ± 1.34	6.16 ± 1.87	13.74 ± 2.16		
	Sham+NeuroEPO n = 6	Ipsi-	3.15 ± 1.20	5.15 ± 0.85	8.92 ± 0.48	5.17 ± 1.75	6.34 ± 1.33
		Contra-	3.11 ± 0.52	5.10 ± 0.57	13.53 ± 3.35		
	TBI+vehicle n = 6	Ipsi-	30.53 ± 8.97 **	25.13 ± 9.27 **	40.88 ± 3.26 ***	37.48 ± 9.50 ****	43.83 ± 6.71 ****
		Contra-	27.43 ± 9.37 **	20.23 ± 4.97 **	30.45 ± 6.67 *		
	TBI+NeuroEPO n = 6	Ipsi-	3.86 ± 1.01 ^##^	8.18 ± 3.21 ^##^	25.06 ± 6.93	6.29 ± 1.54 ^####^	10.72 ± 2.06 ^####^
		Contra-	2.99 ± 0.22 ^##^	6.95 ± 1.41 ^##^	27.10 ± 4.62		
MDA (nmoles of MDA/mg of protein)	Sham+vehicle n = 6	Ipsi-	167.7 ± 12.60	151.2 ± 13.31	142.1 ± 8.84	72.66 ± 13.84	85.41 ± 14.99
		Contra-	142.5 ± 10.75	173.6 ± 31.01	159.2 ± 9.88		
	Sham+NeuroEPO n = 6	Ipsi-	172.2 ± 9.85	171.0 ± 28.28	193.4 ± 34.59	102.4 ± 15.17	143.8 ± 36.78
		Contra-	152.6 ± 14.22	120.4 ± 7.00	155.0 ± 11.19		
	TBI+vehicle n = 6	Ipsi-	554.6 ± 38.2 ****	850.6 ± 16.09 ****	418.2 ± 52.03 ****	213.0 ± 8.01 **	369.9 ± 43.09 ****
		Contra-	402.9 ± 50.0 ****	527.0 ± 86.72 ****	349.2 ± 47.04 **		
	TBI+NeuroEPO n = 6	Ipsi-	190.3 ± 7.12 ^####^	150.4 ± 15.98 ^####^	419.0 ± 38.29 ****	100.1 ± 23.46 ^#^	160.4 ± 44.73 ^####^
		Contra-	136.3 ± 12.1 ^####^	182.8 ± 19.86 ^####^	304.6 ± 59.72 *		

Each value represents the mean ± SEM. Asterisks (*) indicate significant differences compared to baseline values of the same group. Hash symbols (#) indicate significant differences vs. TBI+vehicle group. Statistical analysis was performed using Two-Way ANOVA, followed by Tukey’s post hoc test. **** *p* < 0.0001. * *p* < 0.05, ** *p* < 0.01, *** *p* < 0.001, **** *p* < 0.0001 vs. Sham+vehicle group; ^#^ *p* < 0.05, ^##^ *p* < 0.01, ^###^ *p* < 0.001, ^####^ *p* < 0.0001. DCF, dichlorofluorescein; MDA, malondialdehyde.

**Table 2:** Effects of the intranasal administration of NeuroEPO on the volume of brain structures evaluated by Magnetic Resonance Imaging in rats submitted to severe TBI.

Structure	Side Evaluated	Experimental Group			
		Sham+vehicle n = 4	Sham+NeuroEPO n = 5	TBI+vehicle n = 6	TBI+NeuroEPO n = 6
Cortex	Ipsi-	85.3 ± 4.1	79.1 ± 2.3	65.3 ± 3.2 **	84.0 ± 2.0 ^###^
	Contra-	93.1 ± 4.7	79.2 ± 4.0	63.0 ± 5.4 ***	80.3 ± 2.8 ^#^
Hippocampus	Ipsi-	42.3 ± 0.7	46.1 ± 6.0	29.8 ± 1.5 ***	44.4 ± 0.6 ^###^
	Contra-	44.0 ± 1.4	48.1 ± 6.0	35.8 ± 2.9	34.4 ± 1.8
Amygdala	Ipsi-	23.9 ± 1.3	24.1 ± 0.6	19.5 ± 0.7 *	24.1 ± 1.2 ^##^
	Contra-	24.2 ± 1.1	24.4 ± 1.1	19.4 ± 0.6 *	23.5 ± 0.9 ^#^
Thalamus	Entire	11.5 ± 1.0	12.8 ± 1.0	8.4 ± 0.5 *	13.7 ± 0.2 ^###^
Hypothalamus	Entire	20.5 ± 0.1	20.5 ± 0.4	17.0 ± 0.5 **	20.1 ± 0.8 ^##^

Each value represents the mean ± SEM in mm^3^. Asterisks (*) indicate significant differences vs. Sham+vehicle group. Hash symbols (#) indicate significant differences vs. TBI+vehicle group. Statistical analysis was performed using one-way ANOVA followed by Tukey’s post hoc test. * *p* < 0.05, ** *p* < 0.01, *** *p* < 0.001; ^#^ *p* < 0.05, ^##^ *p* < 0.01, ^###^ *p* < 0.0001.

## Data Availability

The original contributions presented in this study are included in the article. Further inquiries can be directed to the corresponding author(s).

## Abbreviations

The following abbreviations are used in this manuscript:

TBA	Thiobarbituric acid
TRIS	Tris(hydroxymethyl)aminomethane
HEPES	N-2-hydroxyethylpiperazine-N-2-ethanesulfonic acid
PBS	phosphate buffer solution
s.c.	subcutaneous
i.m.	intramuscular
i.p.	intraperitoneal
LFPI	lateral fluid percussion injury model
NS	Neuroscore
TBI	traumatic brain injury
SE	standard error
RM	repeated-measures
